# A cross‐sectional study of low birth satisfaction during the COVID‐19 epidemics' fifth wave

**DOI:** 10.1002/nop2.70026

**Published:** 2024-09-03

**Authors:** Forough Mortazavi, Maryam Mehrabadi

**Affiliations:** ^1^ Non‐Communicable Diseases Research Center Sabzevar University of Medical Sciences Sabzevar Iran; ^2^ MSc of Midwifery, Health Chancellery Sabzevar University of Medical Sciences Sabzevar Iran

**Keywords:** caesarean section, childbirth, COVID‐19, midwifery, nursing, patient satisfaction, postpartum period

## Abstract

**Aim:**

To investigate predictors of low birth satisfaction in a sample of Iranian postpartum women during the COVID‐19 epidemics' fifth wave.

**Design:**

A cross‐sectional study.

**Methods:**

This study was conducted on 676 postpartum women admitted to postpartum wards of Mobini maternity hospital using a convenience sampling method between 2 Aug and 18 September 2021 in Iran. We used the general linear model and multiple linear regression analyses to determine predictors of birth satisfaction.

**Results:**

The mean and standard deviation values of age and education were 28.7 ± 6.6 and 11.1 ± 4.1 (years), respectively. The mean scores of the three scales were as follows: FVC‐19S (14.7 ± 7.5), WHO‐5 (67.5 ± 13.0) and BSS‐R (28.6 ± 7.3). Sixty‐five point nine percent (65.9%) of the participants were multiparous. Overall predictors of low birth satisfaction were emergency caesarean, instrumental birth, episiotomy, Entonox analgesia, low level of well‐being score < 50, fear of COVID‐19, low satisfaction with pregnancy and low satisfaction with spouse's support. The overall proportion of the variance in birth satisfaction explained by all variables is 17.4%. Labor and birth variables explained 12.2% of the variance in birth satisfaction.

**Patient or Public Contribution:**

None.

## 1 INTRODUCTION

Childbirth is a multifaceted and varied experience that elicits a range of emotions, from joy and fulfilment to fear and trauma. According to the World Health Organization, 2018, the aims of intrapartum care should be a healthy mother and also a healthy child. The WHO also recommends positive birth experience as an important objective with regard to all women undergoing labor (World Health Organization, 2018). Birth satisfaction refers to the mother's overall assessment of childbirth and the extent to which she perceived it as a positive and satisfying experience (Karlström et al., 2015). Positive childbirth experience by women is influenced by their preparation for coping with the situation, the support they receive from nurses and midwives (Hosseini Tabaghdehi et al., 2020), and empathy and respectful behaviour on the part of nurses and midwives (Pantoja et al., 2020).

Studies in high‐income countries report that 7%–10% of women have had a negative birth experience (Bell et al., 2018). A number of poor outcomes including postpartum depression, difficulties in mother‐baby bonding, poor maternal care of the baby and abstaining from exclusive breastfeeding have been associated with birth dissatisfaction (Bell et al., 2018). Findings of a study to examine the impact of the COVID‐19 pandemic on birth satisfaction and perceived health care discrimination during childbirth in New York City hospitals on 237 women indicate that women with lower birth satisfaction were more likely to experience higher levels of anxiety, stress and depressive symptoms during the postpartum period (Janevic et al., 2021). Birth dissatisfaction may also increase requests for an elective caesarean for the next birth (Pang et al., 2008) and the likelihood of mothers delaying their next pregnancy (Preis et al., 2020).

Several factors have been found to contribute to dissatisfaction with childbirth including emergency caesarean (Mortazavi & Mehrabadi, 2022; Pang et al., 2008), elective caesarean, higher family income (Pang et al., 2008), use of epidural analgesia (Kempe & Vikström‐Bolin, 2020), induction (Johansson & Finnbogadóttir, 2019), primiparity, low level of well‐being, low satisfaction with pregnancy, severe fear of childbirth, lack of support from partner (Mortazavi & Mehrabadi, 2022) and duration of labor (Demis et al., 2020; Inversetti et al., 2021; Kempe & Vikström‐Bolin, 2020).

The COVID‐19 pandemic has led to increasing rates of anxiety, depression, and stress among women during pregnancy, childbirth, and postpartum (Mariño‐Narvaez et al., 2021; Mollard et al., 2021; Mortazavi & Ghardashi, 2021; Safi‐Keykaleh et al., 2022). Stress was related to constrained antenatal care during the pandemic, worries about being infected with the virus, the possibility of negative effects of the infection on the baby and worry about the health of loved ones (Mortazavi et al., 2021). Fear of COVID‐19 has been found to be a common problem during the pandemic with a significant impact on women's well‐being (Mortazavi & Ghardashi, 2021). It had a statistically significant relationship with depression, suicidal tendencies, and poor psychological quality of life in pregnant women (Ahorsu et al., 2022). The pandemic has also impacted the administrative organization and the practice of nursing and midwifery care provision in the antenatal, intrapartum, and postnatal periods. Factors that might have been impacted include nurses' and midwives' behaviour towards women, more restrictive hospital rules and the likelihood of experiencing a hassle‐free pregnancy. It may also have increased households' economic concerns. Therefore, it would not be unreasonable to expect birth dissatisfaction in women giving birth during COVID‐19 pandemic (Mariño‐Narvaez et al., 2021). Thus, we hypothesize that maternal birth experience and childbirth satisfaction might have been affected by COVID‐19 pandemic. In the present study, we investigated the extent to which fear of COVID‐19, maternal well‐being status, and labor and birth factors predicted lower birth satisfaction during the COVID‐19 epidemics' fifth wave in Sabzevar, Iran.

## 2 MATERIALS AND METHODS

### 2.1 Design, participants and data collection

This cross‐sectional study was conducted from Aug 2 to September 18, 2021. Sampling was done using the convenience method. Women were recruited in the postpartum and surgery wards of Mobini Hospital affiliated with Sabzevar University of Medical Sciences. As a matter of hospital policy, they were usually hospitalized for the first 24 h and 48 h after vaginal delivery and caesarean, respectively. The average annual birth rate in the hospital which was 5898 before the COVID‐19 outbreak, decreased to 5245 in the first year after the outbreak. There were 4020 births in the first 9 months of the second year of the pandemic.

Women who gave birth to a healthy, single, live infant were included in the study. We excluded women undergoing treatment for mental illness, those experiencing severe intra and postpartum complications, individuals with infants admitted to the intensive care unit, those with infants having a 5‐min Apgar score <7, women who delivered infants with congenital malformations, those with preterm births, and women who tested positive for COVID‐19.

A research colleague, who was a midwifery graduate, identified eligible mothers and, if they agreed to participate in the study, presented them with written consent forms and anonymous questionnaires. We trained the midwife on how to present the questionnaire and collect the data. Demographic, social and obstetric information of the mothers were extracted from their medical records and recorded by the midwife. After recording the socio‐demographic information of the women in the questionnaire, the midwife asked women to fill out the scales. The participants received instructions on how to fill out the questionnaires. Additionally, to alleviate women's concerns and worries regarding their infants, the midwife stayed by their side and assisted with newborn care while the women completed the questionnaires. Women who had vaginal deliveries completed the 22‐item study questionnaire on the morning of the day after childbirth when they felt they were ready for the task. In the caesarean group, they filled out the scales on the morning of the second day after birth.

### 2.2 Overview of the setting

During the early pandemic, women could not have a companion in the postpartum ward. Also, childbirth preparatory classes were closed and midwives were reluctant to offer their services as private midwives. But after 1 month the situation improved. Virtual preparatory classes became available and private midwives resumed their services supporting women at birth. The fifth wave of the epidemic, caused by the delta variant, resulted in the highest number of infections and deaths in comparison with previous waves in the country. Although a lockdown was imposed, access to health care facilities was not limited and pregnant women could visit hospitals. They were also allowed to have a private midwife in labor and a companion during postpartum. At this juncture in the COVID‐19 pandemic in Iran, vaccination of pregnant women had not yet been begun but almost all midwives had been infected and all had been vaccinated.

### 2.3 Instruments

A three‐part questionnaire was completed by the midwife. The first part contained questions on socio‐demographic characteristics (including age, education, residency and job). The second part consisted of obstetrical information (such as parity, attending prenatal classes, history of chronic disease, the desirability of pregnancy, poor obstetric history, infant gender, history of abortion and complicated pregnancy). The third part consisted of labor and birth information such as mode of birth, induced or spontaneous labor pain, pain relief method during labor and birth (Entonox, epidural/spinal, nursing support), having a private midwife at birth, gestational age (week), birth weight (gram), admission to birth duration (minute) and episiotomy/tear repair.

The level of satisfaction with pregnancy was assessed based on the extent of health problems experienced during pregnancy using a five‐point Likert scale from 1 to 5 (1 = dissatisfied to 5 = very satisfied). Women's satisfaction with husband's emotional/financial support and marital/sexual satisfaction were evaluated with the same scale. A question on receiving fundal pressure was also included. Women rated their household income as 1 = insufficient or 2 = sufficient (File S1).

#### 2.3.1 The birth satisfaction scale–revised (BSS‐R)

The BSS‐R, created by Hollins Martin & Martin, 2014, is regarded as the best instrument for measuring women's birth satisfaction (Nijagal et al., 2018). The BSS‐R includes 10‐items in three factors: stress experienced during labor, women's personal attributes, and quality of care provision. Participants were asked to rate each item on a four‐point Likert scale which ranges from 0 to 4 (0 = strongly disagree to 4 = strongly agree). The BSS‐R showed acceptable internal consistency (Cronbach's alpha = 0.79). Its total score ranges from 0 to 40 (Hollins Martin & Martin, 2014). The scale was translated into Persian. The validity study of the Persian BSS‐R indicted that it has three dimensions which are identical to those proposed by the developers of the original scale. The reliability of the Persian BSS‐R has been confirmed (Cronbach's alpha = 0.76) (Mortazavi et al., 2020). In the present study, we calculated a Cronbach's alpha value of 0.734.

#### 2.3.2 The World Health Organization's well‐being index (WHO‐5 well‐being index)

The 5‐item World Health Organization Well‐Being Index (WHO‐5) assesses the emotional well‐being of individuals over the preceding 2 weeks. It has five items which are rated using a six‐point Likert scale where zero represents ‘having good feelings at no time’ and five represent ‘having good feelings all the time’ (WHO‐5., 2020). The scale's total score ranges from 0 to 25 which is converted to a scale of 0 to 100. The scale is used in screening programs for depression with a score of 50 as the cut‐off point. Individuals with scores less than 50 should be referred for further assessment. The validity and reliability of the Persian version of WHO‐5 in pregnant women were confirmed and its unidimensionality and reliability was proven (Cronbach's alpha = 0.85) (Mortazavi et al., 2015). In our study, we calculated a Cronbach's alpha value of 0.889.

#### 2.3.3 Fear of COVID‐19 scale (FCV‐19S)

The FCV‐19S is one of the most widely used scales for assessing fear of COVID‐19. It was developed by Ahorsu et al. (2020) for the purpose of assessing fear of COVID‐19. The scale was translated into Persian and the validity study for the scale was conducted using a sample of Iranian students (Ahorsu et al., 2020). It is a unidimensional instrument consisting of seven items rated on a five‐point Likert‐type scale (1–5 points). The total score ranges from 7 to 35 with higher scores indicating a higher level of fear of COVID‐19. A Cronbach's alpha value of 0.88 was reported in the original study on the scale (Ahorsu et al., 2020). In our study, we calculated a Cronbach's alpha value of 0.928.

### 2.4 Data analysis

We used the SPSS version 18 to analyse the data. Descriptive statistics were used to characterize the participants. We evaluated the normal distribution of birth satisfaction scores and other quantitative variables using skewness and kurtosis. Missing data in the WHO‐5 well‐being scale, FCV‐19S and the BSS‐R subscales were substituted with the mean values of the each scale or subscale scores for each participant. The SPSS software was used for this purpose and missing data substitution was done in cases where 20% or less of the data were missing in a scale. We used the general univariate linear model to identify independent variables with a significant impact on birth satisfaction scores. Then, variables with *p* < 0.25 in the univariate linear regression were entered into five separate multiple linear regression analyses by the backward‐LR method. These analyses enabled us to determine the demographic, obstetric, labor and birth, psychological, and overall predictors of birth dissatisfaction. We checked linear regression assumptions. The normality of residuals was verified and collinearity statistics indicated no multicollinearity (tolerance <1 and variance inflation factor <2). The effect sizes for the entire model and main predictors of birth satisfaction scores were calculated. To determine the sample size, we considered the percentage of women in a previous study whose birth satisfaction score was less than the median score of the BSS (Mortazavi & Mehrabadi, 2022). The percentage of such cases was 48%. We calculated the sample size using Cochran's formula (pqz^2^/d^2^) with the confidence level of 95% and a margin of error of 4%. The minimum sample size according to our calculation is estimated to be 599. To address potential sources of bias, we asked women to fill out the questionnaires when they are physically and mentally ready. We also asked them to place the questionnaires in anonymous envelopes before delivering them to our colleagues.

## 3 RESULTS

**FIGURE 1:**
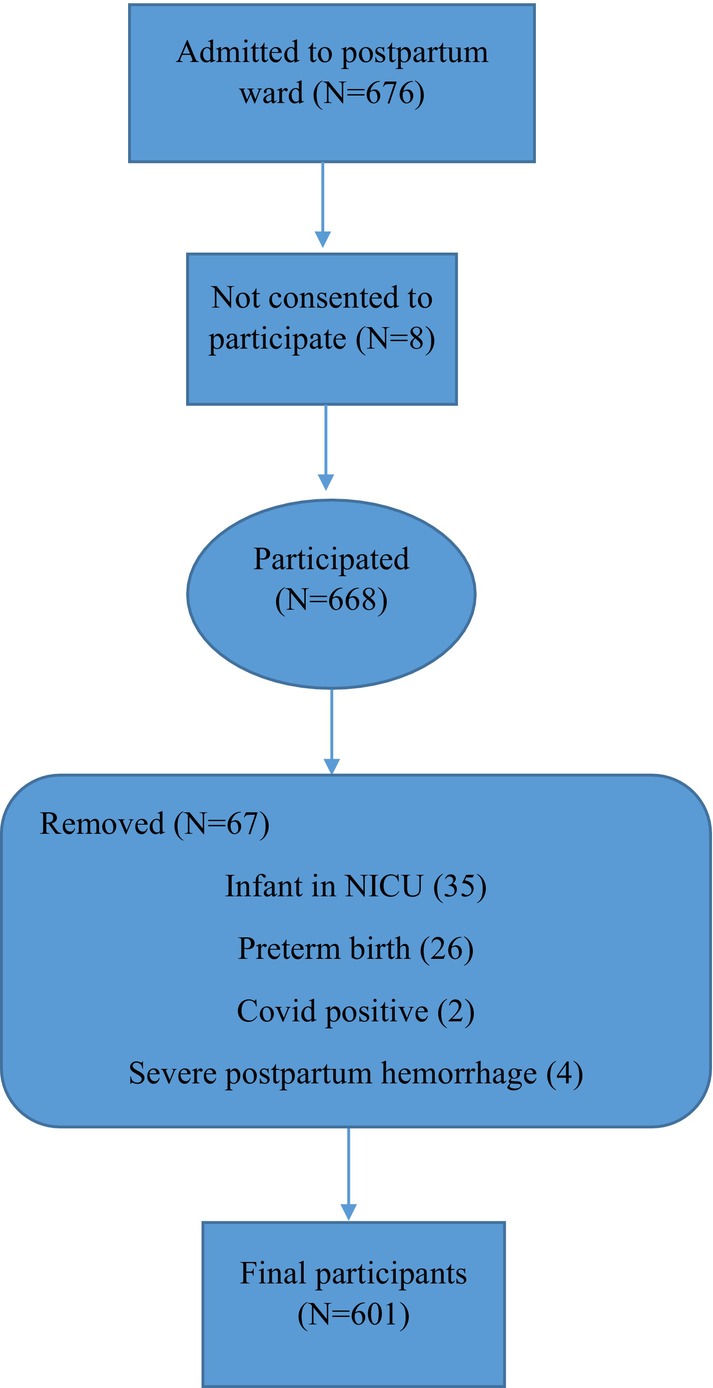
Recruitment process flowchart.

Of the 676 women who gave birth at Mobini hospital during the study period, eight women did not consent to participate in the study. We excluded 67 women from the study, of whom two women were COVID positive, 26 had a preterm birth, 35 women had an infant in the NICU and four women experienced severe postpartum haemorrhage. Overall, 601 women participated in the study (Figure 1). The mean and standard deviation values of socio demographic/obstetric variables were as follows: age (28.7 ± 6.6) (years), education (11.1 ± 4.1) (years), gestational age (39.1 ± 1.2) (weeks), birth weight (3250 ± 465) (grams) and admission to delivery duration (7.2 ± 8.0) (h). The mean scores of the three scales were as follows: FVC‐19S (14.7 ± 7.5), WHO‐5 (67.5 ± 13.0) and BSS‐R (28.6 ± 7.3). Sixty‐five point nine percent (65.9%) of the participants were multiparous. The correlation between birth satisfaction total scores and the duration between admission to hospital and giving birth was 0.247 (*p* < 0.001). Sample characteristics and the results of general linear models for the relationship between the BSS‐R scores and independent variables are presented in Table 1.

**TABLE 1:** Sample characteristics, mean (SD) of birth satisfaction scores, and the results of general linear models on birth satisfaction scores (*N* = 601).

Demographic variables	*N* (%)	M (SD) of birth satisfaction scores^€^	Mean difference (95% CI)	*p*
Age (years)				
≤ 20	70 (11.6)	28.3 ± 7.6	Ref	
21–34	405 (67.4)	28.7 ± 7.3	0.42 (−1.44, 2.27)	0.659
≥ 35	126 (21.0)	28.4 ± 7.2	0.12 (−2.01, 2.25)	0.912
Educational level (years)				
Primary school	94 (15.6)	29.9 ± 6.7	−2.36 (−4.59, −0.13)	0.034*
High school	335 (55.7)	28.8 ± 7.6	−1.15 (−3.19, 0.88)	0.518
University	172 (28.6)	27.6 ± 6.9	Ref	
Job				
Housewife	547 (91.0)	28.8 ± 7.1	Ref	0.086†
Employed	54 (9.0)	27.0 ± 8.7	−1.79 (−3.82, 0.025)	
Household income				
Insufficient	107 (17.8)	27.9 ± 8.6	−0.90 (−2.42, 0.63)	0.249
Sufficient	494 (82.2)	28.8 ± 7.0	Ref	
Resident				
Urban	411 (68.4)	28.1 ± 7.2	−1.49 (−2.74, 0.24)	0.019†, *
Rural	190 (31.6)	29.6 ± 7.3	Ref	
Obstetric variables				
Parity				
Primipara	205 (34.1)	26.9 ± 7.4	−2.62 (−3.83, −1.40)	<0.001***, †
Multipara	396 (65.9)	29.6 ± 7.0	Ref	
History of abortion				
Yes	176 (29.3)	28.3 ± 7.4	−0.38 (−1.66, 0.90)	0.558
No	425 (70.7)	28.7 ± 7.2	Ref	
Infant gender				
Female	315 (52.4)	28.8 ± 6.9	0.48 (−0.69, 1.64)	0.425
Male	286 (47.6)	28.4 ± 7.7	Ref	
Complicated pregnancy				
Yes	123 (20.5)	28.1 ± 8.0	−0.66 (−2.11, 0.79)	0.371
No	478 (79.5)	28.7 ± 7.1	Ref	
Poor obstetric history				
Yes	51 (8.5)	26.9 ± 8.2	−1.86 (−3.95, 0.23)	0.081†
No	550 (91.5)	28.8 ± 7.2	Ref	
Chronic disease				
Yes	62 (10.3)	27.8 ± 8.4	−0.91 (−2.83, 1.01)	0.352
No	539 (89.7)	28.7 ± 7.1	Ref	
Attending prenatal class				
Yes	206 (34.3)	27.8 ± 7.3	−1.27 (−2.49, −0.04)	0.042*, †
No	395 (65.7)	29.0 ± 7.2	Ref	
Labor and Birth				
Gestational age at birth (week)				
< 38	91 (15.1)	29.2 ± 7.9	1.07 (−0.81, 2.96)	0.263
38–40	354 (58.9)	28.7 ± 7.2	0.55 (−0.83, 1.92)	0.438
>40	156 (26.0)	28.1 ± 7.0	Ref	
Birth weight (gr)				
2500–3999	536 (89.2)	28.8 ± 7.2	Ref	
< 2500	24 (4.0)	27.3 ± 8.6	−1.55 (−4.52, 1.43)	0.308
≥ 4000	41 (6.8)	26.3 ± 7.4	−2.50 (−4.80, −0.19)	0.034*, †
Mode of birth				
Elective caesarean	117 (19.5)	30.6 ± 5.9	Ref	
Emergency caesarean	65 (10.8)	23.0 ± 7.9	−7.59 (−9.70, −5.48)	<0.001***, †
Vaginal delivery	391 (65.1)	29.1 ± 7.0	−1.49 (−2.93, −0.05)	0.043*, †
VBAC^£^	11 (1.8)	29.0 ± 5.7	−1.07 (−5.92, 2.70)	0.464
Instrumental delivery	17 (2.8)	24.1 ± 8.1	−6.49 (−10.04, −2.94)	<0.001***, †
Labor pain				
Spontaneous	306 (50.9)	29.8 ± 7.4	Ref	
Induced	179 (29.8)	26.9 ± 7.6	−1.92 (−3.25, −0.60)	0.004**, †
Elective caesarean	116 (19.3)	30.7 ± 5.9	1.86 (0.33, 3.34)	0.018*, †
Having a private midwife				
Yes	150 (25.0)	28.3 ± 7.2	Ref	
No	336 (55.9)	28.0 ± 7.6	−0.30 (−1.70, 1.10)	0.669
Elective caesarean	115 (19.1)	30.7 ± 6.0	−2.34 (−4.10, 0.59)	0.009**
Fundal pressure				
Yes	308 (51.2)	28.4 ± 7.3	−1.99 (−3.57, −0.42)	0.013*, †
No	111 (18.5)	30.4 ± 6.4	Ref	
Caesarean	182 (30.3)	27.9 ± 7.6	−2.48 (−4.19, −0.77)	0.005**
Suturing				
Yes—spontaneous tear	193 (32.1)	29.7 ± 7.1	−1.12 (−3.02, 0.77)	0.245†
Yes—episiotomy	143 (24.8)	27.0 ± 7.3	−3.83 (−5.80, −1.85)	<0.001***, †
Caesarean	182 (30.3)	27.9 ± 7.6	−2.89 (−4.81, −0.98)	0.003**
No	77 (12.8)	30.8 ± 5.9	Ref	
Pain relief method^‡^				
Caesarean‐general analgesia	24 (4.0)	29.2 ± 6.7	−1.16 (−4.38, 2.05,)	0.478
Caesarean‐Spinal analgesia	158 (26.3)	27.7 ± 7.8	−2.63 (−4.41, −0.84)	0.004**, †
Vaginal birth‐Entonox	291 (48.4)	28.3 ± 7.1	−2.01 (−3.62, −0.40)	0.015*, †
Vaginal birth‐Hot water shower/massage/birth ball	22 (3.7)	29.9 ± 7.9	−0.38 (−3.71, 2.96)	0.825
Nothing	106 (17.6)	30.3 ± 6.7	Ref	
Admission to birth duration (hour)				
< 5	289 (48.1)	30.0 ± 6.4	Ref	
≥ 5	312 (51.9)	27.4 ± 7.8	−2.60 (−3.75, −1.46)	<0.001***, †
Psychological variables				
Desirability of pregnancy				
Wanted	378 (62.9)	28.3 ± 7.0	Ref	
Unplanned	74 (12.3)	27.8 ± 9.0	−0.474 (−2.29, 1.34)	0.607
Unwanted	149 (24.8)	29.7 ± 7.0	1.42 (0.04, 2.80)	0.044*, †
Fear of COVID‐19				
Low fear (< the median score)	280 (46.6)	29.3 ± 7.6	Ref	
High fear (≥ the median score)	321 (53.4)	28.0 ± 6.9	−1.27 (−2.44, −0.11)	0.032*, †
Well‐being index (WHO‐5 score)				
< 50	139 (23.1)	26.7 ± 7.1	−2.48 (−3.85, −1.11)	<0.001***, †
≥ 50	462 (76.9)	29.2 ± 7.3	Ref	
Satisfaction with pregnancy				
Low satisfied	133 (22.1)	26.6 ± 8.0	−3.23 (−4.99, −1.46)	<0.001***, †
Moderately satisfied	139 (23.1)	27.8 ± 6.9	−2.00 (−3.75, −0.27)	0.017*, †
Satisfied/very satisfied	329 (54.7)	29.8 ± 6.9	Ref	
Perceived marital/sexual satisfaction				
Low satisfied	46 (7.6)	28.0 ± 9.3	−0.72 (−2.93, 1.49)	0.521
Moderately satisfied	91 (15.1)	28.1 ± 6.7	−0.70 (−2.34, 0.94)	0.401
Satisfied/very satisfied	464 (77.3)	28.8 ± 7.2	Ref	
Satisfaction with spouse's support				
Low satisfied	40 (6.7)	25.7 ± 9.2	−3.15 (−5.49, −0.81)	0.008**, †
Moderately satisfied	74 (12.3)	28.6 ± 7.0	−0.30 (−2.07, 1.48)	0.742
Satisfied/very satisfied	487 (81.0)	28.9 ± 7.1	Ref	

^†^ Variables entered in the multiple linear regression analysis.

^£^ Vaginal birth after caesarean.

^‡^ NVD group was included in the analysis.

^€^ Higher mean scores are indicative of higher birth satisfaction, the highest and lowest possible scores (0–40).

* <0.05.

** <0.01.

*** <0.001.

In Table 2, the results of multiple linear regression analysis for the birth satisfaction scores are presented. Obstetric and labor and birth predictors of birth satisfaction were primiparity [*B* = −2.650, CI (−3.861, −1.439)], birth weight (gr) ≥ 4000 [−2.351 (−4.521, −0.180)], emergency caesarean [−7.332 (−9.276, −5.389)], and episiotomy [−2.466 (−3.853, −1.079)]. Psychological predictors of low birth satisfaction were unwanted pregnancy [2.292 (0.958, 3.625)], Well‐being score < 50 [−1.742 (−3.145, −0.338)], low satisfaction with spouse's support [−2.523 (−4.828, −0.219)], and low [−2.910 (−4.400, −1.419)] and moderate [−2.168 (−3.580, −0.755)] satisfaction with pregnancy. Overall predictors of birth satisfaction were emergency caesarean [−7.463 (−9.310, −5.616)], instrumental delivery [−3.571 (−6.907, −0.235)], episiotomy [−2.227 (−3.591, −0.862)], Entonox analgesia [−1.548 (−2.726, −0.371)], Well‐being score < 50 [−1.812 (−3.146, −0.478)], fear of COVID‐19 [−1.216 (−2.288, −0.144)], low satisfaction with pregnancy −2.539 (−3.952, −1.127) and low satisfaction with spouse's support [−2.419 (−4.598, −0.240)]. The overall proportion of the variance in birth satisfaction explained by all variables is 17.4% (effect size = 0.174). Labor and birth variables explained 12.2% of the variance in birth satisfaction.

**TABLE 2:** Results of multiple linear regression analysis on the birth satisfaction scores.

	Coefficients			Collinearity statistics	
	Beta	B (95.0% CI)	*p*	Tolerance	VIF
Demographic predictors					
Primary school vs. University	0.073	1.464 (−0.280, 3.209)	0.100	0.836	1.196
Urban vs. rural	−0.082	−1.280 (−2.600, 0.041)	0.057	0.891	1.123
Insufficient income	−0.078	−1.490 (−3.068, 0.089)	0.064	0.920	1.086
Obstetrics predictors					
Primiparity	−0.173	−2.650 (−3.861, −1.439)	<0.001***	0.999	1.001
Poor obstetric history	−0.077	−1.998 (−4.057, 0.062)	0.057	0.999	1.001
Labor and birth predictors					
Birth weight (gr) ≥ 4000	−0.082	−2.351 (−4.521, −0.180)	0.034*	0.996	1.004
Instrumental birth	−0.072	−3.170 (−6.600, 0.258)	0.070	0.924	1.083
Emergency caesarean	−0.313	−7.332 (−9.276, −5.389)	<0.001**	0.819	1.221
Induced labor pain	−0.069	−1.102 (−2.360, 0.156)	0.086	0.902	1.109
Episiotomy repair	−0.146	−2.466 (−3.853, −1.079)	0.001**	0.832	1.202
Vaginal birth‐Entonox	−0.078	−1.142 (−2.385, 0.101)	0.072	0.774	1.293
Psychological predictors					
Unwanted pregnancy	0.136	2.292 (0.958, 3.625)	0.001**	0.958	1.044
Well‐being score < 50	−0.101	−1.742 (−3.145, −0.338)	0.015*	0.907	1.103
High fear of COVID‐19 (scores ≥13)	−0.070	−1.016 (−2.151, 0.119)	0.079	0.991	1.009
Low satisfaction with spouse's support	−0.086	−2.523 (−4.828, −0.219)	0.032*	0.963	1.039
Low satisfaction with pregnancy	−0.166	−2.910 (−4.400, −1.419)	<0.001***	0.830	1.205
Moderate satisfaction with pregnancy	−0.126	−2.168 (−3.580, −0.755)	0.003**	0.896	1.116
Overall predictors					
Emergency caesarean (ref: elective caesarean)	−0.319	−7.463 (−9.310, −5.616)	<0.001***	0.853	1.172
Instrumental birth	−0.081	−3.571 (−6.907, −0.235)	0.036*	0.918	1.090
Episiotomy repair	−0.132	−2.227 (−3.591, −0.862)	0.001**	0.809	1.237
Vaginal birth‐Entonox	−0.106	−1.548 (−2.726, −0.371)	0.010*	0.811	1.233
Well‐being score < 50	−0.105	−1.812 (−3.146, −0.478)	0.008**	0.887	1.127
High fear of COVID‐19	−0.083	−1.216 (−2.288, −0.144)	0.026*	0.981	1.019
Low satisfaction with pregnancy	−0.145	−2.539 (−3.952, −1.127)	<0.001***	0.816	1.225
Low satisfaction with spouse's support	−0.083	−2.419 (−4.598, −0.240)	0.030*	0.952	1.051
Primary school vs. university	0.069	1.372 (−0.112, 2.856)	0.070	0.966	1.035
Unwanted pregnancy	0.075	1.259 (−0.028, 2.547)	0.055	0.908	1.101
Moderate satisfaction with pregnancy	−0.076	−1.319 (−2.660, 0.023)	0.054	0.877	1.140
Birth weight (gr) ≥ 4000	−0.070	−2.022 (−4.154, 0.110)	0.063	0.971	1.029

*Note*: Adjusted R square for the first regression analysis = 1.2%, Adjusted R square for the second regression analysis = 3.2%, Adjusted R square for the third regression analysis = 12.2%, Adjusted R square for the fourth regression analysis = 7.5%, Adjusted R square for the fifth regression analysis = 17.4%, method: backward.

* <0.05.

** <0.01.

*** <0.001

We investigated the relationship between the presence of a private midwife during labor and fear of COVID‐19 in the case of women who had a vaginal delivery. Women who were accompanied by a private midwife reported a higher level of fear of COVID‐19 than those without a private midwife (*p* = 0.044). The presence of a private midwife during labor had no relationship with birth satisfaction scores.

The distribution of psychological variables including birth satisfaction, well‐being, and fear of COVID‐19 according to parity indicate that the BSS‐R mean scores was higher in multiparas than primiparas (*p* < 0.001). The FVC‐19S and WHO‐5 mean scores were not different between multiparas and primiparas (*p* > 0.05).

We investigated the predictors of low birth satisfaction in early postpartum. To measure birth satisfaction, there are two scales developed for measuring birth experience: the BSS‐R and the Childbirth Experience Questionnaire (CEQ2) (Walker et al., 2020). We examined both scales and found similar results with regard to their ability to explain birth satisfaction variance (File S2). Table S1 presents descriptive analysis of the CEQ2 and the BSS‐R and their factors. Table S2 presents the correlations between the CEQ2 and the BSS‐R. Table S3 shows the results of general linear models on the CEQ 2 (childbirth experience scores) to disclose labor and birth predictors. The results of general linear models on the CEQ 2 (birth experience scores) to disclose overall predictors is presented in Table S4.

## 4 DISCUSSION

We explored the role of variables related to labor and birth and also psychological variables in birth satisfaction during the COVID‐19 epidemics' fifth wave in Iran. Our results indicate that emergency caesarean, instrumental birth, episiotomy, Entonox analgesia, low well‐being score, high fear of COVID‐19, low satisfaction with pregnancy and a low satisfaction with spouse's support are predictors of lower levels of birth satisfaction. The overall proportion of the variance in birth satisfaction explained by all variables is 17.4%. Labor and birth variables explained 12.2% of the variance in birth satisfaction.

Our results are in line with those of Preis and colleagues (Preis et al., 2021). They found that in the pandemic period, established predictors of low birth satisfaction such as nulliparity, mode of birth, social support, and labor and birth complications explained 35% of the variance in birth satisfaction. According to the same study, pandemic‐related variables including maternal concerns about preparation for birth and restrictions on the number of family members allowed to accompany a birthing mother explained 3% of the variance in birth satisfaction (Preis et al., 2021).

We found that fear of contracting COVID‐19 during the fifth wave of the disease was a predictor of lower birth satisfaction. Previous studies have found that the level of stress experienced during pregnancy and childbirth was significantly associated with birth dissatisfaction (Mariño‐Narvaez et al., 2021; Urbanová et al., 2021). We found no difference in the levels of fear of COVID‐19 between parity or income groups, implying that all mentioned groups of women experienced equal levels of fear of COVID‐19. In contrast, the results of a previous study indicate that fear of COVID‐19 was associated with parity and stage of pregnancy (Giesbrecht et al., 2022).

Our results indicate that birth satisfaction was highest in the elective caesarean group compared to other groups, including vaginal birth, emergency caesarean, and instrumental birth. This suggests that elective caesarean delivery provides greater satisfaction for women. This is not unexpected, given the conventional maternity care provided during labor and birth in our setting. So the aforementioned findings may be explained by delays in providing labor‐delivery‐recovery (LDR) in our setting and by shortcomings in the implementation guidelines promoting physiological vaginal birth, which prohibit fundal pressure and restrict episiotomy. Results from previous studies indicated that caesarean was associated with lower birth satisfaction in comparison with physiological vaginal birth (Inversetti et al., 2021; Janevic et al., 2021; Kahalon et al., 2021; Kempe & Vikström‐Bolin, 2020; Mortazavi & Mehrabadi, 2022; Preis et al., 2021; Urbanová et al., 2021). The difference between our results and other studies may lie in the fact that we compared elective caesarean with other groups, whereas those studies compared caesarean with other groups. In our study, the rate of elective caesarean was 19.3%, which is higher compared to the rate of 13.2% reported in a study conducted on 767 postpartum women in Iran before the pandemic (Mortazavi & Mehrabadi). Similar results were reported in a retrospective cohort study on birth satisfaction in Australia. In this study, the elective caesarean rate increased by 1.7% during the pandemic (from 18.7% to 20.4%) (Trinh et al., 2023).

In our study, a low level of well‐being among women served as a predictor of lower birth satisfaction. A similar result was found in a previous study on birth satisfaction conducted on 736 postpartum women before the pandemic (Mortazavi & Mehrabadi, 2022). Furthermore, the findings of a recent systematic review on the prevalence of postpartum depression during the COVID‐19 pandemic indicate a high rate of postpartum depression (Safi‐Keykaleh et al., 2022).

We found that vaginal birth by episiotomy rather than spontaneous tear is a predictor of low birth satisfaction. In our sample, 24.8 and 32.1 percent of the women experienced episiotomy and spontaneous tear, respectively. Our study was conducted in a mother friendly hospital and so episiotomy is not performed as a routine procedure; however, because it is a training hospital, newly assigned obstetrics residents may perform episiotomy. In a study in Tehran, Iran, vaginal birth by episiotomy accompanied by tear was a predictor of birth satisfaction (Nahaee et al., 2020). In a study in a birth centre in Sweden, an anal sphincter injury was found to be the cause of negative birth experience (Johansson & Finnbogadóttir, 2019).

Our results indicate that receiving Entonox analgesia is a predictor of lower birth satisfaction. Entonox analgesia is typically used for vaginal birth. These findings may be justified by considering that women who request Entonox analgesia expect complete pain relief, an expectation that may not always be met. Our result is in accord with those reported by Fumagalli and colleagues. Their findings indicate that none of the intrapartum interventions was associated with birth satisfaction (Fumagalli et al., 2021).

In our study, having a stressful, hassled pregnancy was a psychological predictor of lower birth satisfaction. Also low satisfaction with spouse's support could predict lower birth satisfaction. These results are in agreement with findings of a previous study on birth satisfaction (Mortazavi & Mehrabadi, 2022). In a study on 225 postpartum women in Khaf, Iran, childbirth experience improved with lower hassle and an increased sense of uplift (Khalife‐Ghaderi et al., 2021). Women with a hassled pregnancy usually experience a high level of stress and perceived insufficient social support (Najjarzadeh et al., 2022). Also, low satisfaction with spouse's support could predict birth satisfaction. These results are in agreement with the findings of previous studies (Khalife‐Ghaderi et al., 2021; Mortazavi & Mehrabadi, 2022). In our study, the rates of low satisfaction with pregnancy and low satisfaction with spouse's support were 22.1% and 6.7%, respectively, which are higher than the rates of 15% and 3.1% reported in a previous study on birth satisfaction in Iran conducted before the pandemic (Mortazavi & Mehrabadi).

We found that giving birth to an infant with high birth weight is a predictor of lower birth satisfaction. High birth weight may cause long labor and higher pain levels which are associated with lower levels of birth satisfaction. The rate of high birth weight (>4000 gr) in our study was 6.8%, which is higher compared to the rate of 4% reported in a previous study on birth satisfaction conducted on postpartum women in Iran before the pandemic (Mortazavi & Mehrabadi). This increase may have been caused by lower levels of physical activity among women during the pandemic.

We found that none of the socio‐demographic variables were related to maternal birth satisfaction. This is in line with previous studies (Fumagalli et al., 2021; Mortazavi & Mehrabadi, 2022). But the results of a study in Africa indicates that informal education of women was a factor associated with women's satisfaction during labor and birth (Demis et al., 2020).

Results from the present study indicate that primiparity is an obstetrical predictor of lower birth satisfaction. This is in agreement with the results of several previous studies (Fumagalli et al., 2021; Nahaee et al., 2020; Urbanová et al., 2021). Satisfaction with childbirth is related to three factors: women's attributes, quality of care received, and stress. Several studies have shown that multiparas experience lower levels of stress and fear of childbirth because of their previous birth experience. Also, giving birth to the first child is usually more difficult than the second or third child. That the satisfaction with current birth is higher in multiparas than primiparas may be the result of their previous experience which decreases their fear of birth and also makes delivery easier and more comfortable.

We found no relationship between the presence of a private midwife during labor and the birth satisfaction score. It seems that the role of private midwives in improving birth satisfaction is not as significant as commonly believed and needs further evaluation. Although childbirth preparatory classes have become increasingly popular among Iranian pregnant women, a study conducted in the country found no significant differences in total satisfaction scores between women with and without regular attendance at childbirth preparation classes (Hassanzadeh et al., 2021). In prenatal visits, pregnant women receive the option to register for free childbirth preparatory classes. During the pandemic, virtual classes were held by midwives who work in private practices or public health centres. After completing the course, participants may opt to have a private midwife during labor, birth and early postpartum. Private midwives are permitted to accompany and support women during active labor, birth, and early postpartum. Most of them do not have permission to perform vaginal exams, delivery of the baby and other interventions. But they usually perform a limited number of nursing support services such as massage, acupressure, and working with the birth ball. Obstetrics residents or midwifery students under the supervision of mentors usually perform the deliveries.

We found that women who had a private midwife during labor and birth reported a higher level of fear of COVID‐19. A possible reason for this may be that during the pandemic, women with higher levels of fear had hired private midwives to receive better care. But because this is a cross‐sectional study, we cannot conclude a causal relationship between the two factors.

### 4.1 Limitations

The main limitation of this study is due to its cross‐sectional design which makes it impossible to establish cause and effect relationships between some variables. We investigated birth satisfaction in the early hours after birth; so, in comparison with studies that were conducted several months after birth, our results are relatively more precise. While we evaluated women's birth satisfaction in early postpartum we did not evaluate women's satisfaction with the nursing support and services received after birth. The latter could have positive or negative effects on overall birth satisfaction. This study was performed using a sample of postpartum women who gave birth in a conventional birth setting. Its findings cannot be generalized to populations who give birth in LDR settings where women are isolated from other parturient and therefore are less worried about contacting COVID‐19.

### 4.2 Implications for future research

Data collection for this study was completed before the vaccination of pregnant women against COVID‐19 had started. We recommend that further studies be undertaken in Iran to explore the effects of vaccination on birth satisfaction. According to our findings, hiring a private midwife was not associated with higher birth satisfaction. We believe that this finding merits further exploration therefore we recommend that qualitative studies be conducted to explore perspectives of women and midwives on the role of private midwives.

## 5 CONCLUSIONS

We found that fear of COVID‐19 is a predictor of lower birth satisfaction. Our findings also indicate that variables related to labor and birth were predictors of birth satisfaction. These variables which were responsible for a large part of the birth satisfaction during the pandemic include the following: emergency caesarean, instrumental birth, Entonox analgesia, episiotomy, low level of well‐being, low satisfaction with pregnancy and low satisfaction with husband's support. Therefore, we recommend that following measures be considered to increase birth satisfaction: interventions to enhance maternal well‐being in late pregnancy, promotion of spouse's support, and interventions to reduce episiotomy, instrumental birth and fear of COVID‐19. Also, policymakers should implement evidence‐based interventions designed to increase birth satisfaction. Health care providers such as obstetricians, midwives and nurses are recommended to plan and conduct intervention studies aimed at increasing birth satisfaction in expectant mothers. Lastly, we recommend that an evidence‐based theoretical model of antenatal and intrapartum support be used in nursing and midwifery instruction and also in clinical practice. The aims of such instruction would be to promote women's satisfaction with pregnancy and also to enhance husband's support.

## AUTHOR CONTRIBUTIONS

MM collected the data and wrote the first draft of the manuscript. FM analysed the data and wrote the final draft of the manuscript. The authors have read and approved the manuscript.

## FUNDING INFORMATION

No specific fund was received.

## CONFLICT OF INTEREST STATEMENT

The authors have no conflict of interest.

## ETHICS STATEMENT

The Ethics Committee of Sabzevar University of Medical Sciences has reviewed and approved this study (approval number: IR.MEDSAB.REC.1399.103). https://ethics.research.ac.ir/EthicsProposalViewEn.php?id=156572 All procedures were performed in accordance with the guidelines of Sabzevar University of Medical Sciences, which is in accordance with the Declaration of Helsinki. Women who consented to participate in the study signed an informed consent form and were assured about the confidentiality of their information.

## Supporting information


File S1.

File S2.

## Data Availability

The datasets used in this study are available from the corresponding author upon reasonable request.
