# Supplementary material for: A cross‐sectional study of low birth satisfaction during the COVID‐19 epidemics' fifth wave
Source: Nurs Open. 2024 Sep 3;11(9):e70026. doi: 10.1002/nop2.70026 (PMC11369488; doi:10.1002/nop2.70026)
Supplement: Supplementary file 1 — File S1. [file NOP2-11-e70026-s002.docx]

**A cross-sectional study of low birth satisfaction during COVID-19 epidemics’ fifth wave**

Dear colleague. Please collect data from the participant’s file carefully.

1. How old is she? ………………. years
2. How many years has she studied? ……………….. years
3. Where does she live?
4. Urban area b. rural area
5. Gestational age at the time of admission ………… week
6. What is her job?
7. Housewife b. employed
8. What is her infant gender?
9. Male b. Female
10. Infant birth weight ………………………… gram
11. How many times has she given birth to a child? Parity = ……
12. How many times has she been pregnant? Gravidity = ……
13. Does she have a chronic disease?

a. Yes b. no

1. Did she have a complicated pregnancy?
2. Yes b. no
3. What is the desirability of pregnancy?
4. Wanted b. unplanned c. unwanted
5. What is the participant’s mode of delivery?
6. Elective cesarean b. emergency cesarean

c. normal vaginal birth d. VBAC e. vacuum delivery

1. Was the labor spontaneous or induced?
2. Spontaneous b. induced c. elective cesarean
3. Did she have a private midwife at birth?
4. No b. yes
5. Did she have a poor obstetric history?
6. No b. yes
7. How long did it take from admission to hospital to giving birth? ………… hours
8. Which analgesic method did she receive in labor?
9. ND.Entonox b. ND. massage c. ND. taking a shower

d. ND. nothing e. ND. epidoural f. ND. spinal

g. cesarean. general anesthesia h. cesarean. spinal

1. Was suturing performed in birth?

a. yes – for spontaneous tear b. yes – for episiotomy repair c. yes – for cesarean d. no

1. Did she participate at prenatal classes?
2. Yes b. no
3. Did she receive fundal pressure in the second stage of birth?
4. Yes b. no

Dear mother. Please response to each question carefully.

1. To what extend are you satisfied with your household income?
2. low level of satisfaction b. satisfaction c. high level of satisfaction
3. To what extend are you satisfied with your pregnancy, given the health problems you encountered during your pregnancy?
4. Not at all b. low satisfied c. moderately satisfied
5. Satisfied e. very satisfied
6. To what extent are you satisfied with your husband’s emotional/financial support?
7. Not at all b. low satisfied c. moderately satisfied
8. Satisfied e. very satisfied
9. To what extent are you satisfied with your marital/sexual relation?
10. Not at all b. low satisfied c. moderately satisfied
11. Satisfied e. very satisfied
