# Supplementary material for: A cross‐sectional study of low birth satisfaction during the COVID‐19 epidemics' fifth wave
Source: Nurs Open. 2024 Sep 3;11(9):e70026. doi: 10.1002/nop2.70026 (PMC11369488; doi:10.1002/nop2.70026)
Supplement: Supplementary file 2 — File S2. [file NOP2-11-e70026-s001.docx]

Table S1. Descriptive analysis of the two scales (the CEQ2 and the BSS-R) and their factors (N=601).

|  | BSS-R | | | | CEQ2 | | | | |
| --- | --- | --- | --- | --- | --- | --- | --- | --- | --- |
|  | Stress | Attribute | Quality Care | BSS-R Total | Capacity | safety | Support | Participation | CEQ2 Total |
| Mean | 11.42 | 4.53 | 12.64 | 28.60 | 21.64 | 17.08 | 16.40 | 12.30 | 67.43 |
| Median | 12.00 | 4.00 | 13.00 | 29.00 | 23.00 | 18.00 | 19.00 | 13.00 | 70.00 |
| Std. Deviation | 4.20 | 2.38 | 2.82 | 7.27 | 5.48 | 4.05 | 4.40 | 2.83 | 13.03 |
| Minimum | 1.00 | .00 | .00 | 1.00 | 8.00 | 6.00 | 5.00 | 4.00 | 23.00 |
| Maximum | 17.00 | 8.00 | 16.00 | 41.00 | 32.00 | 24.00 | 20.00 | 16.00 | 92.00 |

^The Childbirth Experience Questionnaire (CEQ2) includes 22 items in four factors assessing four domains with regard to childbirth experience: own capacity, perceived safety, professional support, and participation. Each item is scored on a five-level Likert scale with a range from zero to four (0- Totally disagree to, 4- Totally agree). The scale’s total score ranges from 0 to 88, with larger scores reflecting higher levels of positive experience.^

Table S2. The correlations between the two scales (the CEQ2 and the BSS-R).

|  | CEQ2 total score | Capacity | Safety | Support | Participation | Fear of Covid -19 |
| --- | --- | --- | --- | --- | --- | --- |
| BSS-R total score | .752** | .670** | .704** | .436** | .479** | -.113** |
| Stress | .685** | .674** | .663** | .355** | .349** | -.086^*^ |
| Quality care | .552** | .398** | .427** | .418** | .507** | -.013 |
| Attributes | .433** | .385** | .472** | .210** | .246** | -.178^**^ |
| Fear of Covid | -.113^**^ | -.101^*^ | -.124^**^ | -.045 | -.077 | 1 |
| ^CEQ2: childbirth Experience Questionnaire , BSS-R: Birth Satisfaction Scale–Revised (BSS-R), *p<0.05, **p<0.01^ | | | | | | |

Table S3. The results of general linear models on the CEQ 2 (childbirth experience scores) (N = 601).

| Model | Unstandardized Coefficients | | Standardized Coefficients | P | 95.0% CI for B | |
| --- | --- | --- | --- | --- | --- | --- |
|  | B | S.E | Beta |  | Lower Bound | Upper Bound |
| Emergency Cesarean | -13.190 | 1.801 | -.315 | <.001 | -16.727 | -9.653 |
| Episiotomy | -4.779 | 1.240 | -.159 | <.001 | -7.214 | -2.343 |
| Entonox | -2.597 | 1.122 | -.100 | .021 | -4.801 | -.392 |
| Birth duration | -2.391 | 1.076 | -.092 | .027 | -4.503 | -.279 |

^a. Dependent Variable: CEQ2 Total score, b: Dependent Variable: labor and birth variables, adjusted R square= 12.7%^

Table S4. The results of general linear models on the CEQ 2 (birth experience scores (N = 601).

| Model | Unstandardized Coefficients | | Standardized Coefficients | P | 95.0% CI for B | |
| --- | --- | --- | --- | --- | --- | --- |
|  | B | S.E | Beta |  | Lower Bound | Upper Bound |
| Emergency Cesarean | -14.046 | 1.793 | -.335 | <.001 | -17.567 | -10.524 |
| Fundal pressure | -2.123 | 1.193 | -.082 | .076 | -4.466 | .220 |
| Episiotomy | -3.305 | 1.323 | -.110 | .013 | -5.904 | -.706 |
| Entonox | -2.565 | 1.137 | -.098 | .024 | -4.799 | -.331 |
| WHO well-being index | -6.268 | 1.155 | -.203 | <.001 | -8.537 | -4.000 |
| Fear of COVID-19 | -1.617 | .971 | -.062 | .097 | -3.525 | .291 |
| Primiparity | -2.958 | 1.135 | -.108 | .009 | -5.186 | -.729 |

^a. Dependent Variable: CEQ2 Total score, b: Dependent Variable: overall variables, adjusted R square= 17.2%^

Walker, K. F., Dencker, A., & Thornton, J. G. (2020). Childbirth experience questionnaire 2: Validating its use in the United Kingdom. *European Journal of Obstetrics & Gynecology and Reproductive Biology: X, 5*, 100097. doi:<https://doi.org/10.1016/j.eurox.2019.100097>
